# Phylogeny of Basal Iguanodonts (Dinosauria: Ornithischia): An Update

**DOI:** 10.1371/journal.pone.0036745

**Published:** 2012-05-22

**Authors:** Andrew T. McDonald

**Affiliations:** Department of Earth and Environmental Science, University of Pennsylvania, Philadelphia, Pennsylvania, United States of America; Raymond M. Alf Museum of Paleontology, United States of America

## Abstract

The precise phylogenetic relationships of many non-hadrosaurid members of Iguanodontia, i.e., basal iguanodonts, have been unclear. Therefore, to investigate the global phylogeny of basal iguanodonts a comprehensive data matrix was assembled, including nearly every valid taxon of basal iguanodont. The matrix was analyzed in the program TNT, and the maximum agreement subtree of the resulting most parsimonious trees was then calculated in PAUP. Ordering certain multistate characters and omitting taxa through safe taxonomic reduction did not markedly improve resolution. The results provide some new information on the phylogeny of basal iguanodonts, pertaining especially to obscure or recently described taxa, and support some recent taxonomic revisions, such as the splitting of traditional “*Camptosaurus*” and “*Iguanodon*”. The maximum agreement subtree also shows a close relationship between the Asian *Probactrosaurus gobiensis* and the North American *Eolambia*, supporting the previous hypothesis of faunal interchange between Asia and North America in the early Late Cretaceous. Nevertheless, the phylogenetic relationships of many basal iguanodonts remain ambiguous due to the high number of taxa removed from the maximum agreement subtree and poor resolution of consensus trees.

## Introduction

Non-hadrosaurid members of Iguanodontia, i.e., basal iguanodonts, are among the most abundant, widespread, and long-studied [1] types of dinosaur [2]. Although they are especially well known from the Early Cretaceous [2], their fossil record extends from the Middle Jurassic (*Callovosaurus* [3]) to the latest Cretaceous (e.g., *Zalmoxes* [4]). Previous phylogenetic analyses included a varying litany of well known basal iguanodonts and revealed the basic arrangement of the various subclades within Iguanodontia, e.g., [4]–[10]. The analysis of Norman [2] has been the most comprehensive dataset available and included a broad taxonomic sample of historical and recently named taxa; however, numerous new taxa have been described since 2004, necessitating a new comprehensive analysis. To investigate the global phylogenetic relationships of basal iguanodonts, I assembled a new data matrix encompassing nearly all valid basal iguanodont taxa. The first iteration of this analysis appeared in McDonald et al. [11] and was subsequently used by Barrett et al. [12]; the second, updated version of the analysis appeared in McDonald et al. [13] and McDonald [14]. A third, updated version of the analysis, which includes additional characters, new taxa, and new information on several taxa, is presented herein.

Institutional Abbreviations: MIWG, Museum of Isle of Wight Geology (Dinosaur Isle Museum), Sandown, UK; NHMUK, Natural History Museum (formerly BMNH, British Museum of Natural History), London, UK.

## Results

### Characters and Taxa Used

The data matrix used in the phylogenetic analysis includes 66 operational taxonomic units (OTUs) (2 outgroups, 61 basal iguanodonts, and 3 representative hadrosaurids) and 135 (70% cranial, 30% postcranial) equally-weighted characters (Supporting Information S1). Forty-six of the OTUs have been examined firsthand by the author; the remaining taxa were coded from the literature or photographs (Supporting Information S2). The data matrix includes all valid basal iguanodont taxa except *Bolong* [15] and *Siamodon* [16] because these taxa have not yet been fully described and have not been inspected firsthand. *Huehuecanauhtlus* [17] is also missing because it was published as this paper was being revised. These three taxa will be included in a future version of the analysis.

Changes made to the data matrix of McDonald et al. [13] and McDonald [14] include modification of character 127 after Barrett et al. [12] and the addition of characters 131–134 (Supporting Information S3). Additional information was added to five taxa: *Valdosaurus* [12], *Barilium* [18], *Jinzhousaurus* [19], *Equijubus* [A. T. McDonald (cranium), and S. Maidment and P. Barrett (postcranium), unpublished data], and *Gilmoreosaurus* [20]. NHMUK R3741 (*cf. Mantellisaurus* in McDonald [21]; considered to represent a possible distinct taxon by Carpenter and Ishida [22]) was coded as a separate OTU, and four new taxa were added: *Delapparentia* [23], *Ratchasimasaurus* [24], *Xuwulong* [25], and *Glishades* [26]. Finally, the coding of character 112 (ilium, morphology of dorsal margin of postacetabular process dorsal to ischial peduncle) for *Iguanacolossus* was changed from 1 (laterally bulging eminence dorsal to ischial peduncle, no modification of dorsal margin) to 2 (mediolaterally thickened dorsal margin compared to dorsal margin above pubic peduncle) upon reconsideration of the morphology of the ilium [following discussion with J. I. Kirkland, pers. comm. Nov. 2011]. Character state 112^1^ is therefore an autapomorphy of *Cedrorestes* (see McDonald et al. [13] for detailed discussion of the ilia of *Iguanacolossus* and *Cedrorestes*).

*Kukufeldia*, from the Grinstead Clay Member of the Tunbridge Wells Sand Formation [11], has been retained as a taxon distinct from *Barilium*, from the Wadhurst Clay Formation [18]. The paucity of overlapping material and absence of exclusively shared morphologies mean that the holotype dentary of *Kukufeldia* cannot be unambiguously referred to *Barilium*. Norman [18] observed that teeth present in the dentary fragment of a specimen referred to *Barilium* (NHMUK R2358; fig. 20 in [18]) resemble the single crown preserved in the holotype dentary of *Kukufeldia* (NHMUK 28660; fig. 4 in [11]), and indeed, the dental morphologies are similar: the marginal denticles are tongue-shaped and bear mammilations, the primary ridge is distally offset, and the crown exhibits parallel and similarly prominent primary and secondary ridges with multiple faint accessory ridges arising from the marginal denticles. However, this combination of features is present in other basal iguanodonts, including *Mantellisaurus atherfieldensis* (NHMUK R5764) and *Iguanodon bernissartensis* (MIWG 1997.55), and thus does not support subjective referral of NHMUK 28660 to *Barilium*.

### First Run, All Characters Unordered: Method and Results

**Figure 1:**
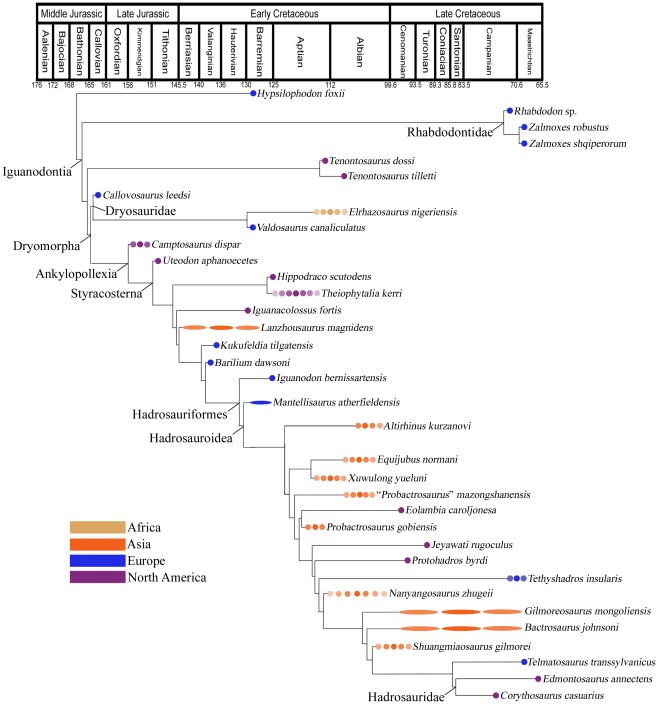
Phylogeny and Temporal and Geographical Occurrences of Basal Iguanodonts. Time-calibrated phylogeny of basal iguanodonts using the maximum agreement subtree of 16,270 MPTs calculated in PAUP. Timescale based upon Walker and Geissman [39]; numerical ages are in millions of years. Uncertainty in taxon ages indicated by lighter circles or ellipses. The branches leading to *Edmontosaurus* and *Corythosaurus* have been extended into the Santonian to reflect the probable age of the oldest known hadrosaurid, the lambeosaurine *Aralosaurus* [9], [40].

The data matrix was analyzed using a traditional search with the tree bisection reconnection algorithm in TNT [27]. Starting trees were Wagner trees with a random seed of 1, and 9,999 replicates were used with 10 trees saved per replication. All characters were treated as unordered. The initial run with all 66 OTUs resulted in 16,270 most parsimonious trees (MPTs) of 377 steps each. The strict consensus tree was very poorly resolved, with nearly the whole of Iguanodontia in an unresolved polytomy; only Rhabdodontidae (*Muttaburrasaurus*, *Rhabdodon*, (*Zalmoxes robustus*, *Z. shqiperorum*)) and *Tenontosaurus* (*T. dossi* plus *T. tilletti*) were resolved as clades. A maximum agreement subtree was calculated in PAUP [28] to examine the underlying topology common to all MPTs; this entailed the deletion of 30 OTUs (Fig. 1). Placement of the various subclades of Iguanodontia (Rhabdodontidae, Dryomorpha, Dryosauridae, Ankylopollexia, Styracosterna, Hadrosauriformes, and Hadrosauroidea) at specific nodes (Fig. 1) follows the definitions of TaxonSearch [29].

### Second Run, 12 Characters Ordered: Method and Results

In the second running of the updated analysis, the search parameters used in TNT remained unchanged. However, 12 multistate characters (10, 14, 20, 25, 46, 67, 81, 82, 83, 100, 127, and 130) were treated as ordered (additive in TNT) using the method of intermediates proposed by Wilkinson [30]. This resulted in 18,610 MPTs of 379 steps. The strict consensus tree was poorly resolved and identical to that obtained by the first run, in which all characters were unordered.

The matrix was then analyzed in the program TAXEQ3 [31] to search for taxonomic equivalents that would allow safe taxonomic reduction. This test indicated that five OTUs, “*Camptosaurus*” *valdensis*, *Draconyx*, NHMUK R8676, *Delapparentia*, and *Glishades*, could be safely deleted. Rerunning the analysis after deletion of those five OTUs produced 28,230 MPTs of 379 steps. The strict consensus cladogram was only slightly more resolved than that produced before safe taxonomic reduction. There was a polytomy at the base of Iguanodontia that included Rhabdodontidae (with the same internal topology as before), *Tenontosaurus*, *Callovosaurus*, *Dryosaurus*, *Kangnasaurus*, and a clade with the topology (*Dysalotosaurus*, (*Elrhazosaurus*, *Valdosaurus*)); this clade was also recovered by Barrett et al. [12]. More derived iguanodontians (Ankylopollexia) were arrayed in a vast polytomy; the only resolution within Ankylopollexia was a clade with the topology (*Bactrosaurus*, (*Shuangmiaosaurus*, *Tanius*, *Telmatosaurus*, *Claosaurus*, *Lophorhothon*, *Hadrosaurus*, *Edmontosaurus*, *Corythosaurus*)).

## Discussion

Monophyletic Camptosauridae and Iguanodontidae were not recovered by any iteration of the analysis, as in some previous analyses [2], [5], [6], [10] but in contrast to others in the case of Iguanodontidae [7], [8]. The results also reinforce several recent taxonomic revisions and phylogenetic hypotheses. *Uteodon aphanoecetes* is more derived than *Camptosaurus dispar* in the maximum agreement subtree (Fig. 1), supporting its removal from *Camptosaurus* [14]. The phylogeny supports the removal of many specimens and species from traditional “*Iguanodon*”; *Kukufeldia tilgatensis* [11], *Barilium dawsoni* [32], *Iguanodon bernissartensis*, and *Mantellisaurus atherfieldensis* [33] comprise a succession of more derived taxa near the base of Hadrosauriformes in the agreement subtree (Fig. 1).

The agreement subtree presents relationships that could carry intriguing paleobiogeographical implications if they are supported by additional analyses. The close relationship between *Probactrosaurus gobiensis* from China and *Eolambia* from Utah in the maximum agreement subtree (Fig. 1) fits in with evidence from other vertebrate groups of the establishment of a connection between Asia and North America in the latest Early Cretaceous and earliest Late Cretaceous [34]. The agreement subtree also includes a close relationship between the Early Cretaceous North American basal styracosternans *Hippodraco* and *Theiophytalia* in both trees (Fig. 1; also found in [13]).

The phylogeny of Iguanodontia presents numerous ghost lineages, some quite long (Fig. 1). The longest ghost lineages, those of Rhabdodontidae (∼95 million years) and *Tenontosaurus* (∼55 million years), occur near the base of Iguanodontia and are due to the relatively extreme antiquity of the most basal members of the less inclusive clades Dryomorpha (the dryosaurid *Callovosaurus*), Ankylopollexia (*Camptosaurus*), and Styracosterna (*Uteodon*), as also noted by Norman [2] and Weishampel et al. [4]. The dryosaurid affinity of *Callovosaurus*, first suggested by Ruiz-Omeñaca et al. [3], pulls the origin of Ankylopollexia into the Callovian and extends the ghost lineages of Rhabdodontidae and *Tenontosaurus* farther back into the Middle Jurassic. It appears that the origin of Iguanodontia lies in the latter half of the Middle Jurassic.

There are also numerous ghost lineages among basal hadrosauroids, including a very long one (∼40 million years) leading to *Tethyshadros*. However, these ghost lineages are probably artificially lengthened by the poorly constrained ages of many Asia taxa, especially *Nanyangosaurus*, *Gilmoreosaurus*, *Bactrosaurus*, and *Shuangmiaosaurus* (Fig. 1). Better resolution on the ages of these taxa will likely reduce the ghost lineages leading to basal hadrosauroids for which more precise age data exist, such as *Jeyawati* [10], *Protohadros* [5], and *Tethyshadros* [35].

### Inclusion of Poorly Known Taxa

It is clear from the lack of resolution in the strict consensus trees and the high number of OTUs deleted from the agreement subtree that many basal iguanodonts acted as wildcard taxa in the phylogenetic analysis. Doubtless this is due to the extremely fragmentary nature of many specimens; numerous taxa are known from a single very incomplete specimen or even a single element (e.g., *Owenodon*, *Cedrorestes*, *Osmakasaurus*, *Kukufeldia*, *Delapparentia*, *Penelopognathus*, *Ratchasimasaurus*, *Glishades*). However, such taxa might present useful phylogenetic information and should not be excluded *a priori*, but only after rigorous application of safe taxonomic reduction and strict reduced consensus methods [36]–[38]. For example, even though it is known from only a complete femur, *Callovosaurus* is found to be a dryosaurid in the agreement subtree (Fig. 1), supporting the affinity proposed by Ruiz-Omeñaca et al. [3]; this has the effect of pulling the origins of other basal iguanodontian clades and of Iguanodontia itself into the Middle Jurassic. This enlightening result would not have been obtained had *Callovosaurus* been excluded *a priori*.

### Conclusions and Prospectus

The phylogenetic positions of many basal iguanodont taxa are highly unstable; the calculation of an agreement subtree leads to greater resolution, but at the price of deleting numerous taxa. This paper may be regarded as a steppingstone towards more detailed macroevolutionary studies of basal iguanodonts. Resolution of basal iguanodont relationships will require additional fossils of previously known and new taxa and additional characters. Additional taxa will be added to the data matrix in the near future; this fourth version will be used to further investigate basal iguanodont phylogeny, paleobiogeography, character evolution, diversity, and the effect of geological bias on those results.

## Methods

For the sake of clarity, the phylogenetic analysis methods are described above with their results (see Results). Several different techniques have been employed, and it is better to present each method and its results together to illustrate more clearly and effectively how each method affected the phylogenetic results.

## Supporting Information

Supporting Information S1 **Data Matrix.**
(XLS)

Supporting Information S2 **Iguanodont Specimen List.**
(XLS)

Supporting Information S3 **Character List and References.**
(DOC)

## References

[1] Mantell G (1825). Notice on the *Iguanodon*, a newly discovered fossil reptile, from the sandstone of Tilgate Forest, in Sussex.. Philosophical Transactions of the Royal Society of London.

[2] Norman DB, Weishampel DB, Dodson P, Osmólska H (2004). Basal Iguanodontia.. The Dinosauria: Second Edition.

[3] Ruiz-Omeñaca JI, Pereda Suberbiola X, Galton PM, Carpenter K (2006). *Callovosaurus leedsi*, the earliest dryosaurid dinosaur (Ornithischia: Euornithopoda) from the Middle Jurassic of England.. Horns and beaks: ceratopsian and ornithopod dinosaurs.

[4] Weishampel DB, Jianu C-M, Csiki Z, Norman DB (2003). Osteology and phylogeny of *Zalmoxes* (n.g.), an unusual euornithopod dinosaur from the latest Cretaceous of Romania.. Journal of Systematic Palaeontology.

[5] Head JJ (1998). A new species of basal hadrosaurid (Dinosauria, Ornithischia) from the Cenomanian of Texas.. Journal of Vertebrate Paleontology.

[6] Norman DB (2002). On Asian ornithopods (Dinosauria: Ornithischia). 4. *Probactrosaurus* Rozhdestvensky, 1966.. Zoological Journal of the Linnean Society.

[7] Kobayashi Y, Azuma Y (2003). A new iguanodontian (Dinosauria: Ornithopoda) from the Lower Cretaceous Kitadani Formation of Fukui Prefecture, Japan.. Journal of Vertebrate Paleontology.

[8] You H-L, Luo Z-X, Shubin NH, Witmer LM, Tang Z-L (2003). The earliest-known duck-billed dinosaur from deposits of late Early Cretaceous age in northwest China and hadrosaur evolution.. Cretaceous Research.

[9] Sues H-D, Averianov A (2009). A new basal hadrosauroid dinosaur from the Late Cretaceous of Uzbekistan and the early radiation of duck–billed dinosaurs.. Proceedings of the Royal Society B: Biological Sciences.

[10] McDonald AT, Wolfe DG, Kirkland JI (2010). A new basal hadrosauroid (Dinosauria: Ornithopoda) from the Turonian of New Mexico.. Journal of Vertebrate Paleontology.

[11] McDonald AT, Barrett PM, Chapman SD (2010). A new basal iguanodont (Dinosauria: Ornithischia) from the Wealden (Lower Cretaceous) of England.. Zootaxa.

[12] Barrett PM, Butler RJ, Twitchett RJ, Hutt S (2011). New material of *Valdosaurus canaliculatus* (Ornithischia: Ornithopoda) from the Lower Cretaceous of southern England.. Special Papers in Palaeontology.

[13] McDonald AT, Kirkland JI, DeBlieux DD, Madsen SK, Cavin J (2010). New basal iguanodonts from the Cedar Mountain Formation of Utah and the evolution of thumb-spiked dinosaurs.. PLoS ONE.

[14] McDonald AT (2011). The taxonomy of species assigned to *Camptosaurus* (Dinosauria: Ornithopoda).. Zootaxa.

[15] Wu W, Godefroit P, Hu D (2010). *Bolong yixianensis* gen. et sp. nov.: a new iguanodontoid dinosaur from the Yixian Formation of western Liaoning, China.. Geology and Resources.

[16] Buffetaut E, Suteethorn V (2011). A new iguanodontian dinosaur from the Khok Kruat Formation (Early Cretaceous, Aptian) of northeastern Thailand.. Annales de Paléontologie.

[17] Ramírez-Velasco AA, Benammi M, Prieto-Márquez A, Ortega JA, Hernández-Rivera R (2012). *Huehuecanauhtlus tiquichensis*, a new hadrosauroid dinosaur (Ornithischia: Ornithopoda) from the Santonian (Late Cretaceous) of Michoacán, Mexico.. Canadian Journal of Earth Sciences.

[18] Norman DB (2011). On the osteology of the lower Wealden (Valanginian) ornithopod *Barilium dawsoni* (Iguanodontia: Styracosterna).. Special Papers in Palaeontology.

[19] Wang X, Pan R, Butler RJ, Barrett PM (2010). The postcranial skeleton of the iguanodontian ornithopod *Jinzhousaurus yangi* from the Lower Cretaceous Yixian Formation of western Liaoning, China.. Earth and Environmental Science Transactions of the Royal Society of Edinburgh.

[20] Prieto-Márquez A, Norell MA (2010). Anatomy and relationships of *Gilmoreosaurus mongoliensis* (Dinosauria: Hadrosauroidea) from the Late Cretaceous of Central Asia.. American Museum Novitates.

[21] McDonald AT (2012). The status of *Dollodon* and other basal iguanodonts (Dinosauria: Ornithischia) from the Lower Cretaceous of Europe.. Cretaceous Research.

[22] Carpenter K, Ishida Y (2010). Early and “Middle” Cretaceous iguanodonts in time and space.. Journal of Iberian Geology.

[23] Ruiz-Omeñaca JI (2011). *Delapparentia turolensis* nov. gen et sp., a new iguanodontoid dinosaur (Ornithischia: Ornithopoda) from the Lower Cretaceous of Galve (Spain).. Estudios Geológicos.

[24] Shibata M, Jintasakul P, Azuma Y (2011). A new iguanodontian dinosaur from the Lower Cretaceous Khok Kruat Formation, Nakhon Ratchasima in northeastern Thailand.. Acta Geologica Sinica-English Edition.

[25] You H, Li D, Liu W (2011). A new hadrosauriform dinosaur from the Early Cretaceous of Gansu Province, China.. Acta Geologica Sinica-English Edition.

[26] Prieto-Márquez A (2010). *Glishades ericksoni*, a new hadrosauroid (Dinosauria: Ornithopoda) from the Late Cretaceous of North America.. Zootaxa.

[27] Goloboff PA, Farris JS, Nixon KC (2008). TNT, a free program for phylogenetic analysis.. Cladistics.

[28] Swofford DL (2005). PAUP* Phylogenetic Analysis Using Parsimony (*and Other Methods). Version 4.0 beta 10.

[29] Sereno PC (2005). Stem Archosauria version 1.0, TaxonSearch.. http://www.taxonsearch.org/Archive/stem-archosauria-1.0.php.

[30] Wilkinson M (1992). Ordered versus unordered characters.. Cladistics.

[31] Wilkinson M (2001). TAXEQ3: software and documentation. Department of Zoology, The Natural History Museum, London.. http://www.nhm.ac.uk/research-curation/research/projects/software/mwphylogeny.html.

[32] Norman DB (2010). A taxonomy of iguanodontians (Dinosauria: Ornithopoda) from the lower Wealden Group (Cretaceous: Valanginian) of southern England.. Zootaxa.

[33] Paul GS, Carpenter K (2006). Turning the old into the new: a separate genus for the gracile iguanodont from the Wealden of England.. Horns and beaks: ceratopsian and ornithopod dinosaurs.

[34] Cifelli RL, Kirkland JI, Weil A, Deino AL, Kowallis BJ (1997). High-precision ^40^Ar/^39^Ar geochronology and the advent of North America's Late Cretaceous terrestrial fauna.. Proceedings of the National Academy of Sciences.

[35] Dalla Vecchia FM (2009). *Tethyshadros insularis*, a new hadrosauroid dinosaur (Ornithischia) from the Upper Cretaceous of Italy.. Journal of Vertebrate Paleontology.

[36] Kearney M, Clark JM (2003). Problems due to missing data in phylogenetic analyses including fossils: a critical review.. Journal of Vertebrate Paleontology.

[37] Wiens JJ (2003). Incomplete taxa, incomplete characters, and phylogenetic accuracy: is there a missing data problem?. Journal of Vertebrate Paleontology.

[38] Butler RJ, Upchurch P (2007). Highly incomplete taxa and the phylogenetic relationships of the theropod dinosaur *Juravenator starki*.. Journal of Vertebrate Paleontology.

[39] Walker JD, Geissman JW (2009). 2009 GSA geologic time scale.. GSA Today.

[40] Godefroit P, Alifanov V, Bolotsky Y (2004). A re-appraisal of *Aralosaurus tuberiferus* (Dinosauria, Hadrosauridae) from the Late Cretaceous of Kazakhstan.. Bulletin de l'Institut Royal des Sciences Naturelles de Belgique, Sciences de la Terre.

