# Supplementary material for: Phylogeny of Basal Iguanodonts (Dinosauria: Ornithischia): An Update
Source: PLoS One. 2012 May 22;7(5):e36745. doi: 10.1371/journal.pone.0036745 (PMC3358318; doi:10.1371/journal.pone.0036745)
Supplement: Supporting Information S3 — Character List and References. (DOC) [file pone.0036745.s003.doc]

**S3, Character List and References**

**Institutional Abbreviations**

**AMNH**, American Museum of Natural History, New York, NY, USA; **ANSP**, Academy of Natural Sciences, Philadelphia, PA, USA; **BELUM**, Ulster Museum (National Museums of Northern Ireland), Belfast, UK; **CEUM**, College of Eastern Utah Prehistoric Museum, Price, UT, USA; **CM**, Carnegie Museum of Natural History, Pittsburgh, PA, USA; **DMNH**, Denver Museum of Nature and Science, Denver, CO, USA; **HERM**, Hull and East Riding Museum, Hull, UK; **IRSNB**, Institut royal des Sciences naturelles de Belgique, Brussels, Belgium; **IVPP**, Institute of Vertebrate Paleontology and Paleoanthropology, Beijing, China; **MIWG**, Museum of Isle of Wight Geology (Dinosaur Isle Museum), Sandown, UK; **MNHN**, Muséum national d’Histoire naturelle, Paris, France; **MSM**, Arizona Museum of Natural History (formerly Mesa Southwest Museum), Mesa, AZ, USA; **NHMUK**, Natural History Museum (formerly BMNH, British Museum of Natural History), London, UK; **OXFUM**, Oxford University Museum of Natural History, Oxford, UK; **PIN**, Palaeontological Institute, Moscow, Russia; **QM**, Queensland Museum, South Brisbane, Australia; **SDSM**, South Dakota School of Mines and Technology, Rapid City, SD, USA; **SMU**, Southern Methodist University Shuler Museum of Paleontology, Dallas, TX, USA; **UMNH**, Natural History Museum of Utah, Salt Lake City, UT, USA; **USNM**, National Museum of Natural History, Washington, DC, USA; **YPM**, Yale Peabody Museum of Natural History, New Haven, CT, USA.

**Character List**

Description of characters used in the phylogenetic analysis of Iguanodontia. Characters are numbered 0–134 in the format of TNT. Abbreviations refer to specific skeletal elements or regions (e.g., PD1 is predentary character 1): PD, predentary; DT, dentary; SU, surangular; ANG, angular; PM, premaxilla; MX, maxilla, LAC, lacrimal, PRF, prefrontal; PO, postorbital; JG, jugal; QJ, quadratojugal; QU, quadrate; SQ, squamosal; BC, braincase; T, teeth; AX, axial column; PC, pectoral girdle; FL, forelimb; PV, pelvic girdle; HL, hind limb. Characters are polarized with respect to the outgroup *Lesothosaurus diagnosticus*.

**Mandibular**

0. Predentary, overall shape of oral portion in dorsal view: subtriangular, comes to a point without a distinct rostral portion (0); arcuate, rounded rostrolateral corners (1); subrectangular, squared corners and straight, well demarcated rostral portion (2) (modified from Weishampel *et al*. 2003, character 18; and Prieto-Márquez *et al.* 2006b, character 5). PD1

1. Predentary, directions of lateral margins of lateral processes relative to each other in dorsal and ventral views: divergent (0); parallel (1). PD2

2. Predentary, morphology of ventromedial process: undivided (0); bifurcated (1) (Weishampel *et al.* 2003, character 20). PD3

3. Predentary, dorsomedial process: absent (0); present (1). PD4

4. Predentary, denticles: absent (0); present (1) (Weishampel *et al*. 2003, character 19). PD5

5. Predentary, denticle morphology: large, conical median denticle with one or two prominent conical denticles of subequal size adjacent to the median denticle on both sides and smaller, tab-like denticles on lateral processes (0); rostrocaudally compressed prong-like denticles that increase in size towards the midline of the predentary (1); rostrocaudally compressed prong-like denticles of equal size (2) (modified from Prieto-Márquez 2010b, characters 25 and 27) PD6

6. Predentary, grooves on either side of midline on rostral surface, extending ventrolaterally to dorsomedially: absent (0); present (1). PD7

7. Dentary, orientation of symphysis relative to lateral margin of dentary: rostrolateral to caudomedial (medial edge of symphysis and lateral margin of dentary diverge in dorsal view) (0); parallel (1) (Prieto-Márquez *et al.* 2006b, character 10). DT1

8. Dentary, orientation of symphyseal region in lateral view: ventral surface of symphyseal region visible in lateral view, articulation site for ventromedial process of predentary faces ventolaterally (0); ventral surface of symphyseal region not visible in lateral view, articulation site for ventromedial process of predentary faces ventrally (1). DT2

9. Dentary, diastema: absent (0); present (1). DT3

10. Dentary, shape of tooth row in dorsal view: bowed medially at mid-length (0); bowed medially along caudal half (1); straight (2) (modified from Prieto-Márquez *et al.* 2006b, character 8). DT4

11. Dentary, shape of tooth row in lateral view: straight (0); concave (1); convex (2). DT5

12. Dentary, orientation of tooth row relative to lateral surface of dentary: convergent rostrally and caudally (0); convergent rostrally and divergent caudally (1). DT6

13. Dentary, morphology of tooth alveoli: alveoli shaped by dentary teeth (0); alveoli with parallel vertical walls (1) (Norman 2002, character 33). DT7

14. Dentary, caudal-most extent of tooth row: rostral to base of coronoid process (0); medial to coronoid process but still rostral to longitudinal axis of the process (1); even with longitudinal axis of the coronoid process (2); caudal to longitudinal axis of the coronoid process but still rostral to the caudal margin of the process (3); caudal to the base of the coronoid process (4) (modified from You *et al.* 2003b, character 29). DT8

15. Dentary, shape in lateral or medial view: tapers rostrally (0); dorsal and ventral margins are parallel (1); deepens rostrally (2) (Norman 2004, character 22). DT9

16. Dentary, morphology of ventral margin of rostral ramus leading to the predentary articulation: straight (0); ventral margin inflected ventrally, such that it curves gently towards the predentary articulation and symphysis (1); ventral margin curves dorsally towards symphysis (2). DT10

17. Dentary, bulge along ventral margin directly ventral to the base of the coronoid process: absent (0); present (1) (modified from Prieto-Márquez 2010b, character 41). DT11

18. Dentary, bulge on the lateral surface ventral to the coronoid process that gives rise to the process: absent (0); present (1) (modified from Prieto-Márquez 2010b, character 46). DT12

19. Dentary, platform between the tooth row and the coronoid process: absent, tooth row curves into base of coronoid process (0); present (1) (modified from Norman 2002, character 26). DT13

20. Dentary, orientation of coronoid process: caudally inclined (0); vertical (1); rostrally inclined (2) (modified from Prieto-Márquez *et al.* 2006b, character 7). DT14

21. Dentary, expansion of dorsal end of coronoid process: absent (0); present (1). DT15

22. Dentary, expansion of dorsal end of coronoid process, location: along rostral edge only (0); along rostral and caudal edges (1) (modified from McDonald *et al.* 2010b, character 33). DT16

23. Dentary, position of greatest rostrocaudal width of expanded coronoid process: ventral to apex (0); at apex (1). DT17

24. Surangular, surangular foramen: present (0); absent (1) (modified from Weishampel *et al.* 1993, character 27). SU1

25. Surangular, external mandibular fenestra: large, open fenestra between dentary, surangular, and angular (0); small foramen (“accessory foramen”) on surangular near suture with dentary (1) absent (2) (modified from Kobayashi & Azuma 2003, character 15). SU2

26. Surangular, shape of contact with angular in lateral view: inclined rostrodorsal to caudoventral (0); sinuous (1); horizontal (2). SU3

27. Angular, exposure in lateral view: present, groove on ventral margin of surangular for articulation with angular (0); absent, articulation with surangular occurs on the medial surface of that bone (1) (modified from Norman 2002, character 28). ANG1

**Cranial**

28. Premaxilla, morphology of rostral margin in dorsal view: premaxillae not transversely expanded, snout comes to a point (0); premaxillae laterally expanded, snout squared (1). PM1

29. Premaxilla, tooth alveoli: present (0); absent (1). PM2

30. Premaxilla, ventral inflection: absent, oral margin even with ventral margin of maxilla (0); present, oral margin projects farther ventrally than ventral margin of maxilla (1) (modified from Norman 2002, character 2). PM3

31. Premaxilla, morphology of caudolateral corner of oral margin in lateral view: in contact with maxilla (0); free and gently curved (1); free and angular (2). PM4

32. Premaxilla, everted rim on lateral edge of oral margin: absent (0); present (1) (modified from Weishampel *et al.* 1993, character 3). PM5

33. Premaxilla, denticles on oral margin: absent (0); present (1) (modified from Weishampel *et al.* 2003, character 7). PM6

34. Premaxilla, denticle morphology: one large conical denticle adjacent to interpremaxillary suture on each premaxilla (0); two large, rostrocaudally elongate denticles on each premaxilla (1); four or more conical denticles of similar size on each premaxilla (2); three rostrally projecting denticles that decrease in size laterally (3). PM7

35. Premaxilla, morphology of caudal ramus of ventrolateral process: tapers to a point (0); dorsoventrally expanded (1) (modified from Prieto-Márquez 2010b, character 71). PM8

36. Premaxilla, contact with lacrimal: absent (0); present (1) (Weishampel *et al.* 2003, character 8). PM9

37. Premaxilla, contact with prefrontal: absent (0); present (1). PM10

38. External naris, position: confined to area immediately above oral margin of premaxilla (0); extends caudally so as to lie dorsal to maxilla (1) (modified from Weishampel *et al.* 2003, character 2). NA1

39. Maxilla, rostrodorsal process: absent (0); present (1) (modified from Prieto-Márquez *et al.* 2006b, character 17). MX1

40. Maxilla, direction of rostroventral process: rostrally directed (0); rostroventrally curved (1). MX2

41. Maxilla, ventral margin of tooth row in lateral view: straight (0); concave (1). MX3

42. Maxilla, shape in dorsal view: bowed medially (0); straight for most of length (1); bowed laterally (2). MX4

43. Maxilla, shape of tooth row in ventral view: medially bowed, with rostral and caudal ends curving laterally (0); bowed laterally (1); straight (2). MX5

44. Maxilla, shape of ascending process: rostrocaudally narrow and hook-like (0); rostrocaudally broad and subtriangular (1). MX6

45. Maxilla, jugal process morphology: dorsally concave, rostrodorsally to caudoventrally inclined shelf, scarf contact with jugal (0); sinuous shelf, scarf contact with jugal (1); caudolaterally projecting jugal process, “finger-in-recess” contact with jugal (2); mediolaterally broad, flat surface against which jugal abuts (3) (modified from Norman 2002, character 15). MX7

46. Maxilla, antorbital fossa, extent in lateral view: occupies most of lateral surface of ascending process (0); rostrocaudally elongate, elliptical depression restricted to caudal half of ascending process (1); small semicircular depression restricted to caudal margin of ascending process (2); antorbital fossa not visible in lateral view (3). MX8

47. Lacrimal, concave ventral margin to form part of antorbital fenestra: present (0); absent (1). LAC1

48. Lacrimal, morphology of rostral ramus: tapers to a point (0); blocky and angular (1); bifurcated (2); dorsoventrally expanded (3). LAC2

49. Lacrimal, morphology of ventral ramus: tapers to a point (0); blocky and angular (1); rounded (2). LAC3

50. Lacrimal, contact with nasal: present (0); absent (1) (Norman 2002, character 12). LAC4

51. Prefrontal, morphology of nasal process: tapering, finger-like projection (0); dorsoventrally broad, mediolaterally compressed plate (1). PRF1

52. Postorbital, shape of caudal end of squamosal process that overlaps the lateral surface of the squamosal: tapers to a point (0); rounded (1); bifurcated (2) (modified from Prieto-Márquez 2010b, character 132). PO1

53. Jugal, articulation with ectopterygoid: present (0); absent (1) (Head 1998, character 6). JG1

54. Jugal, morphology of portion of maxillary process that overlaps maxilla: tapers at rostral ends of maxillary and lacrimal contacts, with slightly convex ventral margin and slightly concave dorsal margin (0); subrectangular with parallel dorsal and ventral margins (1); tapers with sinuous dorsal and ventral margins (2); dorsoventrally expanded (3); dorsoventrally expanded to form part of rostral margin of orbit (4) (modified from Norman 2002, character 14). JG2

55. Jugal, large neurovascular foramen at base of postorbital process on medial surface: absent (0); present (1). JG3

56. Jugal, shape of free ventral margin caudal to maxillary and ectopterygoid contacts: straight (0); sinuous, jugal dorsoventrally expanded ventral to infratemporal fenestra (1); sinuous with striated, caudally-directed flange that projects caudal to jugal-quadratojugal contact (2); angular, with prominent ventrally-directed flange ventral to infratemporal fenestra (3); dorsoventrally narrow and strap-like, with convex ventral margin and concave dorsal margin that are parallel (4) (modified from Norman 2002, character 16). JG4

57. Jugal, contribution of caudal ramus to ventral margin of infraorbital fenestra: partial, quadratojugal also forms part of margin (0); caudal ramus forms entire ventral margin of infraorbital fenestra (1) (Weishampel *et al.* 2003, character 11). JG5

58. Quadratojugal, quadratojugal foramen: absent (0); present (1) (Weishampel *et al.* 2003, character 17). QJ1

59. Quadrate, quadratojugal notch in lateral wing: absent (0); present (1) (McDonald *et al.* 2010b, character 16). QU1

60. Quadrate, shape of notch in lateral wing: semicircular (0); broad and crescentic (1) (modified from Prieto-Márquez *et al.* 2006b, character 40). QU2

61. Quadrate, paraquadrate foramen: absent, caudal margin of quadratojugal contacts entire rostral margin of quadrate along the contact surface (0); present, gap between portion of caudal margin of quadratojugal and rostral margin of quadrate (1). QU3

62. Quadrate, overall shape in lateral or medial view: curved gently caudally along entire length (0); straight for much of dorsoventral length, curved caudally near dorsal end (1); straight (2). QU4

63. Quadrate, shape of dorsal condyle: subtriangular, broad rostral margin and tapers to a point caudally (0); subrectangular (1); D-shaped, broadest along lateral profile (2) QU5

64. Quadrate, shape of ventral condyle: rostrocaudally narrow and mediolaterally broad, with larger lateral condylar surface and lateral and medial condyles on same plane (0); asymmetrical with enlarged, more ventrally situated lateral condyle (1). QU6

65. Squamosal, morphology of postorbital process dorsal to *M. adductor mandibulae externus superficialis* origin site: gently convex (0); mediolaterally compressed and blade-like (1). SQ1

66. Squamosal, orientation of caudomedial process: curved rostromedially (0); curved caudomedially (1); straight and medially directed (2) (modified from Prieto-Márquez *et al.* 2006b, character 45). SQ2

67. Squamosal, relationship of right and left squamosals on skull roof: widely separated by parietal (0); separated by only a narrow band of the parietal (1); in broad contact with each other (2) (Horner *et al.* 2004, character 63). SQ3

68. Frontal, participation in dorsal orbital rim: present (0); absent (1) (Norman 2002, character 19). BC1

69. Supraoccipital, contribution to foramen magnum: present (0); absent, excluded by exoccipitals (1) (You *et al.* 2003b, character 23). BC2

70. Supraoccipital, morphology of supraoccipital-exoccipital contact: straight suture that meets squamosal (0); ventrolateral corner of supraoccipital is inset into exoccipital so that supraoccipital is locked between exoccipitals (1) (Horner *et al.* 2004, character 66). BC3

71. Supraoccipital, inclination of caudal surface: caudal surface rostrally inclined (0); caudal surface vertical (1) (modified from Horner *et al.* 2004, character 65). BC4

72. Exoccipital-Opisthotic, paroccipital process shape: dorsoventrally expanded distally (0); pendant (1) (Weishampel *et al.* 2003, character 13). BC5

73. Exoccipital-Opisthotic, paroccipital process orientation of pendant distal portion: straight and ventrally directed (0); curved rostrally (1) (Horner *et al.* 2004, character 62). BC6

74. Basioccipital, orientation of occipital condyle: caudoventrally directed (0); caudally directed (1) (modified from Prieto-Márquez 2010b, character 152). BC7

75. Basioccipital, rostrocaudally directed groove extending along ventral surface: absent (0); present (1). BC8

76. Basioccipital, rostrocaudally directed, sharply defined ridge between basal tubera: absent (0); present (1). BC9

77. Basisphenoid, surface between basipterygoid processes: smooth (0); transverse, sharply defined ridge between basipterygoid processes (1); ventrally directed prong between basipterygoid processes (2) (modified from Gates & Sampson 2007, characters 78 and 79). BC10

78. Basisphenoid, orientation of basipterygoid processes: ventolaterally directed and rostrally inclined (0); ventrolaterally directed and caudally curved (1) (modified from Prieto-Márquez *et al.* 2006b, character 83). BC11

79. Foramen magnum, composition of ventral margin: caudomedial surfaces of left and right exoccipitals and dorsal margin of basioccipital (0); left and right exoccipitals only (1) (modified from Weishampel *et al.* 1993, character 24). BC12

**Dental**

80. Dentary teeth, intercrown spaces: present (0); absent (1) (You *et al*. 2003b, character 32). T1

81. Dentary teeth, morphology of marginal denticles: tongue-shaped with smooth edges (0); tongue-shaped with mammillated edges (1); reduced to small mammillated papillae (2) (modified from Norman 2002, character 31). T2

82. Dentary teeth, number of replacement teeth per tooth position: one (0); two (1); three (2) (modified from Weishampel *et al.* 1993, character 32). T3

83. Dentary teeth, number of teeth per tooth position forming part of occlusal plane: one (0); two (1); three (2) (modified from Norman 2002, character 39). T4

84. Dentary teeth, shape of crown in lingual view: mesiodistally broad, oblong, shield-like surface (0); mesiodistally narrow and diamond-shaped (1) (modified from Norman 2002, character 29). T5

85. Dentary teeth, ridges on lingual surface of crown: absent (0); present (1). T6

86. Dentary teeth, position of primary ridge: mesially offset (0); distally offset (1); no offset, primary ridge divides the lingual side of the crown into equal halves (2) (modified from You *et al.* 2003, character 39). T7

87. Dentary teeth, number and morphology of ridges on lingual surface of crown: prominent primary ridge and multiple separate faint accessory ridges on both sides of it (0); prominent primary ridge and multiple evenly-spaced accessory ridges on either side such that entire lingual surface is corrugated (1); parallel and similarly prominent primary and secondary ridges with multiple faint accessory ridges arising from marginal denticles (2); primary ridge and a single less prominent accessory ridge on either side (3); primary ridge and a single mesial accessory ridge (4); primary ridge only (5). T8

88. Maxillary teeth, intercrown spaces: present (0); absent (1). T9

89. Maxillary teeth, number of teeth per tooth position forming part of occlusal plane: one (0); two (1). T10

90. Maxillary teeth, ridges on labial surface of crown: absent (0); present (1). T11

91. Maxillary teeth, primary ridge position and morphology: distally offset (0); no offset, primary ridge divides the labial side of the crown into equal halves (1) (modified from You *et al.* 2003b, character 36). T12

92. Maxillary teeth, number and morphology of ridges on labial surface of crown: primary ridge with multiple parallel accessory ridges on either side (0); multiple ridges of similar size, such that primary ridge cannot be distinguished (1); primary ridge and only mesial accessory ridges (2); primary ridge only (3). T13

**Postcranial (Axial)**

93. Axis, morphology of axial neural spine in lateral view: caudodorsally sloping process (0); dorsally expanded process (1). AX1

94. Cervical vertebrae, opsisthocoely of centra: slightly opisthocoelous, flat or slightly convex cranial face (0); deeply opisthocoelous, hemispherical cranial face protrudes beyond ventral and dorsal surfaces of centrum and has a smooth, rounded surface (1). AX2

95. Ossified epaxial tendons, arrangement along dorsal, sacral, and caudal vertebrae: longitudinally arranged (0); double-layered lattice (1) (Weishampel *et al.* 2003, character 42). AX3

**Postcranial (Appendicular)**

96. Sternal, caudolateral process: absent (0); present (1) (modified from Kobayashi & Azuma 2003, character 23). PC1

97. Sternal, caudomedial process: absent (0); present (1). PC2

98. Sternal, shape of main body in dorsal or ventral view, excluding caudolateral process if present: convex medially and concave laterally (0); convex medially and straight laterally (1). PC3

99. Scapula, dorsal margin of scapular shaft: straight (0); convex (1). PC4

100. Scapula, expansion of caudal end: gently convex expansion along caudodorsal margin, caudoventral margin tapers into hook-like flange (0); caudal end paddle-shaped, dorsal and ventral margins of scapula diverge towards caudal end (1); caudal margin of scapula straight, dorsal and ventral margins are parallel approaching caudal margin of scapula and meet caudal margin at nearly right angles (2). PC5

101. Scapula, shape of acromion process in lateral view: subtriangular (0); cranial margin of process is convex (1); low, rounded protuberance (2). PC6

102. Scapula, acromion process orientation: dorsally directed (0); laterally directed (1) (Norman 2002, character 44). PC7

103. Humerus, shape of deltopectoral crest: distal margin rounded and merges gradually with the lateral margin of the humeral shaft (0); distal margin angular and merges abruptly with the lateral margin of the humeral shaft (1) (modified from Weishampel *et al.* 1993, character 37). FL1

104. Manus, digit I: present (0); absent (1) (Norman 2002, character 51). FL2

105. Manus, ungual of digit I, shape: claw-like (0); conical (1) (modified from Norman 2002, character 52). FL3

106. Manus, arrangement of metacarpals II-IV: spreading (0); closely appressed (1) (You *et al.* 2003b, character 49). FL4

107. Manus, unguals of digits II and III, shape: claw-like (0); flattened and hoof-like (1) (Norman 2002, character 53). FL5

108. Manus, digit III, number of phalanges: four (0); three (1) (Weishampel *et al.* 2003, character 53). FL6

109. Ilium, preacetabular process, cranial end: rounded (0); horizontal boot (1). PV1

110. Ilium, preacetabular process, twisting along its length such that the dorsal surface of the ilium becomes the lateral surface of the cranial end of the process: absent (0); present (1) (modified from Weishampel *et al.* 2003, character 56). PV2

111. Ilium, dorsal margin above pubic and ischial peduncles and acetabulum: straight (0); convex (1); sinuous, convex above pubic peduncle and concave above ischial peduncle (2) (modified from Weishampel *et al.* 2003, character 55). PV3

112. Ilium, morphology of dorsal margin of postacetabular process dorsal to ischial peduncle: smooth surface (0); laterally bulging eminence dorsal to ischial peduncle, no modification of dorsal margin (1); mediolaterally thickened dorsal margin compared to dorsal margin above pubic peduncle (2); thickened and laterally-bulging everted rim along dorsal margin (3); laterally-projecting, non-pendant shelf continuous with dorsal margin of ilium (4); pendant supraacetabular process continuous with dorsal margin of ilium (5) (modified from Norman 2002, character 56). PV4

113. Ilium, postacetabular process, shape in lateral view: rounded with break in slope along dorsal margin, parallel dorsal and ventral margins (0); tapers to a point with break in slope along dorsal margin, forming a distinct platform for the origin of *M. iliocaudalis* (1); tapers with no break in slope along dorsal margin (2); subrectangular with no break in slope (3) (modified from Norman 2002, character 57). PV5

114. Ilium, postacetabular process: caudally directed (0); process curves dorsally along its entire length, such that the both the dorsal and ventral margins curve dorsally (1). PV6

115. Pubis, cranial expansion of cranial pubic process: absent, dorsal and ventral margins parallel (0); present, dorsal and ventral margins diverge distally (1) (modified from Norman 2002, character 58). PV7

116. Pubis, dorsal margin of cranial pubic process: straight (0); concave (1). PV8

117. Pubis, caudal pubic process: approximately equal in length to ischium (0); shorter than ischium (1) (Norman 2002, character 59). PV9

118. Pubis, caudal pubic process, morphology of distal end: rounded (0); tapers to a point (1). PV10

119. Ischium, morphology of shaft: curved caudally (0); curved cranially (1); straight (2) (modified from Norman 2002, character 60). PV11

120. Ischium, morphology of distal end: rounded expansion (0); cranially expanded boot (1); bluntly truncated (2) (modified from Prieto-Márquez *et al.* 2006b, character 126). PV12

121. Femur, curvature of shaft: distal half of shaft curved caudally (0); distal half of shaft straight (1) (Norman 2002, character 62). HL1

122. Femur, groove on caudal aspect of femoral head: present (0); absent (1) (Winkler *et al.* 1997, character 25). HL2

123. Femur, morphology of fourth trochanter: pendant (0); broad and triangular (1); curved, laterally compressed eminence (2) (Norman 2002, character 63). HL3

124. Femur, location of fourth trochanter: arises on proximal half of femoral shaft (0); arises at midshaft of femur (1) (Weishampel *et al.* 2003, character 69). HL4

125. Femur, location of insertion scar of *M. caudifemoralis longus*: extends from fourth trochanter onto medial surface of femoral shaft (0); widely separated from fourth trochanter, restricted to medial surface of femoral shaft (1) (Ruiz-Omeñaca *et al.* 2006). HL5

126. Femur, intercondylar extensor groove: absent (0); present (1) (Weishampel *et al.* 2003, character 70). HL6

127. Femur, intercondylar extensor groove: broad, shallow, V-shaped, edges of groove meet at greater than 90 degrees to one another (0); tight, deep, V-shaped, edges of groove meet at less than 90 degrees (1); deep, narrow, U-shaped, partially enclosed by slight expansion of medial condyle (2); deep, U-shaped, partially enclosed by expansion of medial and lateral condyles (3); canal fully enclosed by fusion of lateral and medial condyles (4) (modified from Norman 2002, character 64; McDonald *et al*. 2010c, character 127; Barrett *et al*. 2011, character 127). HL7

128. Femur, intercondylar flexor groove: completely open, U-shaped trough (0); partially closed by lateral inflation of medial condyle (1) (modified from Weishampel *et al.* 2003, character 71). HL8

129. Pes, prominent lip extending proximodorsally from dorsal margins of phalangeal proximal articulation facets: present (0); absent (1). HL9

130. Pes, morphology of unguals on digits II-IV: dorsoventrally flattened, but elongate and pointed (0); dorsoventrally flattened and elongate, but with blunt truncated tips (1); hoof-like shape (2) (modified from Norman 2002, character 67). HL10

**New Characters**

131. Premaxilla, transverse ridge of thickened bone caudal to oral margin, separated from the oral margin by a deep sulcus bearing vascular foramina: absent (0); present (1) (modified from Prieto-Márquez 2010a, character 63). PM11

132. Ilium, brevis fossa, transverse width: narrow (0); very broad and expanding in width towards its caudal margin such that it appears triangular in dorsal or ventral view (1) (Barrett *et al*. 2011, character 132). PV13

133. Femur, deep cleft separating the greater and lesser trochanters: present (0); absent, lesser trochanter is closely appressed to the proximal end of the femur (1) (modified from Barrett *et al*. 2011, character 134). HL11

134. Metatarsals III and IV, deep caudolateral notch on MT III for the reception of a prominent process of MT IV: absent (0); present (1) (modified from Barrett *et al*. 2011, character 135). HL12

**Supplemental References (there is some overlap with the article itself)**

Allain, R. & Pereda Suberbiola, X. 2003 Dinosaurs of France. *Comptes Rendus Palevol* **2**,

27–44.

Barrett, P. M. 1996 The first known femur of *Hylaeosaurus armatus* and re-identification of

ornithopod material in The Natural History Museum, London. *Bulletin of the Natural History Museum of London (Geology)* **52**, 115–118.

Barrett, P. M., Butler, R. J., Twitchett, R. J. & Hutt, S. 2011 New material of *Valdosaurus*

*canaliculatus* (Ornithischia: Ornithopoda) from the Lower Cretaceous of southern England. *Special Papers in Palaeontology* **86**, 131–163.

Barrett, P. M., Butler, R. J., Wang X.-L. & Xu X. 2009 Cranial anatomy of the iguanodontoid

ornithopod *Jinzhousaurus yangi* from the Lower Cretaceous Yixian Formation of China. *Acta Palaeontologica Polonica* **54**, 35–48.

Bartholomai, A. & Molnar, R. E. 1981 *Muttaburrasaurus*, a new iguanodontid (Ornithischia:

Ornithopoda) dinosaur from the Lower Cretaceous of Queensland. *Memoirs of the*

*Queensland Museum* **20**, 319–349.

Brill, K. & Carpenter, K. 2006 A description of a new ornithopod from the Lytle Member of

the Purgatoire Formation (Lower Cretaceous) and a reassessment of the skull of

*Camptosaurus*. In *Horns and Beaks: Ceratopsian and Ornithopod Dinosaurs* (ed. K. Carpenter), pp. 49–67. Bloomington: Indiana University Press.

Brown, B. 1914 *Corythosaurus casuarius*, a new crested dinosaur from the Belly River

Cretaceous, with provisional classification of the family Trachodontidae. *Bulletin of the American Museum of Natural History* **33**, 559–564.

Brown, B. 1916 *Corythosaurus casuarius*: skeleton, musculature and epidermis. *Bulletin of the*

*American Museum of Natural History* **35**, 709–716.

Burton, D., Greenhalgh, B. W., Britt, B. B., Kowallis, B. J., Elliott, W. S. Jr. & Barrick, R. 2006.

New radiometric ages from the Cedar Mountain Formation, Utah and the Cloverly Formation, Wyoming: implications for contained dinosaur faunas. *Geological Society of America Abstracts with Programs* **38**, 52.

Carpenter, K. & Ishida, Y. 2010 Early and “Middle” Cretaceous iguanodonts in time and space.

*Journal of Iberian Geology* **36**, 145–164.

Carpenter, K. & Wilson, Y. 2008 A new species of *Camptosaurus* (Ornithopoda: Dinosauria)

from the Morrison Formation (Upper Jurassic) of Dinosaur National Monument, Utah,

and a biomechanical analysis of its forelimb. *Annals of the Carnegie Museum* **76**, 227–263.

Carpenter, K., Dilkes, D. & Weishampel, D. B. 1995 The dinosaurs of the Niobrara Chalk

Formation (Upper Cretaceous, Kansas). *Journal of Vertebrate Paleontology* **15**, 275–297.

Cooper, M. R. 1985 A revision of the ornithischian dinosaur *Kangnasaurus coetzeei* Haughton,

with a classification of the Ornithischia. *Annals of the South African Museum* **95**, 281–317.

Dalla Vecchia, F. M. 2009a *Telmatosaurus* and the other hadrosauroids of the Cretaceous

European Archipelago. An update. *Natura Nascosta* **39**, 1–18.

Dalla Vecchia, F. M. 2009b *Tethyshadros insularis*, a new hadrosauroid dinosaur

(Ornithischia) from the Upper Cretaceous of Italy. *Journal of Vertebrate Paleontology* **29**, 1100–1116.

DiCroce, T. & Carpenter, K. 2001 New ornithopod from the Cedar Mountain Formation

(Lower Cretaceous) of eastern Utah. In *Mesozoic Vertebrate Life* (eds. D. H. Tanke & K. Carpenter), pp. 183–196. Bloomington: Indiana University Press.

Forster, C. A. 1990 The postcranial skeleton of the ornithopod dinosaur *Tenontosaurus tilletti*.

*Journal of Vertebrate Paleontology* **10**, 273–294.

Galton, P. M. 1974 The ornithischian dinosaur *Hypsilophodon* from the Wealden of the Isle of

Wight. *Bulletin of the British Museum (Natural History) Geology* **25**, 1–152.

Galton, P. M. 1981 *Dryosaurus*, a hypsilophodontid dinosaur from the Upper Jurassic of North

America and Africa. Postcranial Skeleton. *Paläontologische Zeitschrift* **55**, 271–312.

Galton, P. M. 1983 The cranial anatomy of *Dryosaurus*, a hypsilophodontid dinosaur from the

Upper Jurassic of North America and East Africa, with a review of hypsilophodontids from the Upper Jurassic of North America. *Geologica et Palaeontologica* **17**, 207–243.

Galton, P. M. 2009 Notes on Neocomian (Lower Cretaceous) ornithopod dinosaurs from

England – *Hypsilophodon*, *Valdosaurus*, “*Camptosaurus*”, “*Iguanodon*” – and referred specimens from Romania and elsewhere. *Revue de Paléobiologie, Genève* **28**, 211–273.

Galton, P. M. & Powell, H. P. 1980 The ornithischian dinosaur *Camptosaurus prestwichii*

from the Upper Jurassic of England. *Palaeontology* **23**, 411–443.

Galton, P. M. & Taquet, P. 1982 *Valdosaurus*, a hypsilophodontid dinosaur from the Lower

Cretaceous of Europe and Africa. *Geobios* **15**, 147–159.

Garrison Jr., J. R., Brinkman, D., Nichols, D. J., Layer, P., Burge, D. & Thayn, D. 2007 A

multidisciplinary study of the Lower Cretaceous Cedar Mountain Formation, Mussentuchit Wash, Utah: a determination of the paleoenvironment and paleoecology of the *Eolambia caroljonesa* dinosaur quarry. *Cretaceous Research* **28**, 461–494.

Gates, T. A. & Sampson, S. D. 2007 A new species of *Gryposaurus* (Dinosauria:

Hadrosauridae) from the late Campanian Kaiparowits Formation, southern Utah, USA. *Zoological Journal of the Linnean Society* **151**, 351–376.

Gilmore, C. W. 1909 Osteology of the Jurassic reptile *Camptosaurus*, with a revision of the

species of the genus, and descriptions of two new species. *Proceedings of the United States National Museum* **36**, 197–332.

Gilmore, C. W. 1933 On the dinosaurian fauna of the Iren Dabasu Formation. *Bulletin of the*

*American Museum of Natural History* **67**, 23–78.

Gilpin, D., DiCroce, T. & Carpenter, K. 2006 A possible new basal hadrosaur from the Lower

Cretaceous Cedar Mountain Formation of eastern Utah. In *Horns and Beaks: Ceratopsian and Ornithopod Dinosaurs* (ed. K. Carpenter), pp. 79–89. Bloomington: Indiana University Press.

Godefroit, P., Codrea, V. & Weishampel, D. B. 2009 Osteology of *Zalmoxes shqiperorum*

(Dinosauria, Ornithopoda), based on new specimens from the Upper Cretaceous of Nǎlaţ-Vad (Romania). *Geodiversitas* **31**, 525–553.

Godefroit, P., Li H. & Shang C.-Y. 2005 A new primitive hadrosauroid dinosaur from the

Early Cretaceous of Inner Mongolia (P. R. China). *Comptes Rendus Palevol* **4**, 697–705.

Godefroit, P., Dong Z.-M., Bultynck, P., Li H. & Feng L. 1998 New *Bactrosaurus*

(Dinosauria: Hadrosauroidea) material from Iren Dabasu (Inner Mongolia, P. R. China). *Bulletin de l’Institut Royal des Sciences Naturelles de Belgique, Sciences de la Terra* **68** (Supplement), 3–70.

Head, J. J. 1998 A new species of basal hadrosaurid (Dinosauria, Ornithischia) from the

Cenomanian of Texas. *Journal of Vertebrate Paleontology* **18**, 718–738.

Head, J. J. 2001 A reanalysis of the phylogenetic position of *Eolambia caroljonesa*

(Dinosauria, Iguanodontia). *Journal of Vertebrate Paleontology* **21**, 392–396.

Hicks, J. F., Brinkman, D. L., Nichols, D. J. & Watabe, M. 1999 Paleomagnetic and palynologic

analyses of Albian to Santonian strata at Bayn Shireh, Burkhant, and Khuren Dukh, eastern Gobi Desert, Mongolia. *Cretaceous Research* **20**, 829–850.

Hooley, R. W. 1925 On the skeleton of *Iguanodon atherfieldensis* sp. nov., from the Wealden

Shales of Atherfield (Isle of Wight). *Quarterly Journal of the Geological Society of London* **81**, 1–61.

Horner, J. R., Weishampel, D. B. & Forster, C. A. 2004 Hadrosauridae. In *The Dinosauria:*

*Second Edition* (eds. D. B. Weishampel, P. Dodson & H. Osmólska), pp. 438–463. Berkeley: University of California Press.

Janensch, W. 1955 Der ornithopode *Dysalotosaurus* der Tendaguruschichten.

*Palaeontographica Supplement* **7**, 105–176.

Jiang, B. & Sha, J. 2006 Late Mesozoic stratigraphy in western Liaoning, China: a review.

*Journal of Asian Earth Sciences* **28**, 205–217.

Kirkland, J. I. 1998 A new hadrosaurid from the upper Cedar Mountain Formation (Albian-

Cenomanian: Cretaceous) of eastern Utah - the oldest known hadrosaurid (lambeosaurine?). In *Lower and Middle Cretaceous Terrestrial Ecosystems* (eds. S. G. Lucas, J. I. Kirkland & J. W. Estep). *New Mexico Museum of Natural History and Science Bulletin* **14**, 283–295.

Kobayashi, Y. & Azuma, Y. 2003 A new iguanodontian (Dinosauria: Ornithopoda) from the

Lower Cretaceous Kitadani Formation of Fukui Prefecture, Japan. *Journal of Vertebrate Paleontology* **23**, 166–175.

Kowallis, B. J., Christiansen, E. H., Deino, A. L., Peterson, F., Turner, C. E., Kunk, M. J. &

Obradovich, J. D. 1998 The age of the Morrison Formation. *Modern Geology* **22**, 235–260.

Langston Jr., W. 1960 The vertebrate fauna of the Selma Formation of Alabama, Part VI: the

dinosaurs. *Fieldiana: Geology Memoirs* **8**, 319–360.

Lü, J. 1997 A new Iguanodontidae (*Probactrosaurus mazongshanensis* sp. nov.) from

Mazongshan Area, Gansu Province, China. In *Sino-Japanese Silk Road Dinosaur Expedition* (ed. Z. Dong), pp. 27–47. Beijing: China Ocean Press.

Marsh, O. C. 1893 The skull and brain of *Claosaurus*. *American Journal of Science, Series 3*

**45**, 83–86.

Mateus, O. & Antunes, M. T. 2001 *Draconyx loureiroi*, a new Camptosauridae (Dinosauria,

Ornithopoda) from the Late Jurassic of Lourinhã, Portugal. *Annales de Paléontologie* **87**, 61–73.

McDonald, A. T. 2011 The taxonomy of species assigned to *Camptosaurus* (Dinosauria:

Ornithopoda). *Zootaxa* **2783**, 52–68.

McDonald, A. T. 2012 The status of *Dollodon* and other basal iguanodonts (Dinosauria:

Ornithischia) from the Lower Cretaceous of Europe. *Cretaceous Research* **33**, 1–6.

McDonald, A. T., Barrett, P. M. & Chapman, S. D. 2010a A new basal iguanodont (Dinosauria:

Ornithischia) from the Wealden (Lower Cretaceous) of England. *Zootaxa* **2569**, 1–43.

McDonald, A. T., Wolfe, D. G. & Kirkland, J. I. 2010b A new basal hadrosauroid (Dinosauria:

Ornithopoda) from the Turonian of New Mexico. *Journal of Vertebrate Paleontology* **30**, 799–812.

McDonald, A. T., Kirkland, J. I., DeBlieux, D. D., Madsen, S. K., Cavin, J., Milner, A. R. C. &

Panzarin, L. 2010c New basal iguanodonts from the Cedar Mountain Formation of Utah and the evolution of thumb-spiked dinosaurs. *PLoS ONE* **5(11)**, e14075.

Meng, Z.-F. 1994. Magnetostratigraphy of Lower Cretaceous (Hekou Group) near Lanzhou,

Gansu. *Chinese Journal of Geophysics* **1994-S2**.

Molnar, R. E. 1996 Observations on the Australian ornithopod dinosaur, *Muttaburrasaurus*.

*Memoirs of the Queensland Museum* **39**, 639–652.

Norman, D. B. 1980 On the ornithischian dinosaur *Iguanodon bernissartensis* from Belgium.

*Mémoires Institut Royal des Sciences Naturelles de Belgique* **178**, 1–103.

Norman, D. B. 1986 On the anatomy of *Iguanodon atherfieldensis* (Ornithischia: Ornithopoda).

*Bulletin de l’Institut Royal des Sciences Naturelles de Belgique* **56**, 281–372.

Norman, D. B. 1993 Gideon Mantell’s “Mantel-piece”: the earliest well-preserved ornithischian

dinosaur. *Modern Geology* **18**, 225–245.

Norman, D. B. 1998 On Asian ornithopods (Dinosauria: Ornithischia). 3. A new species of

iguanodontid dinosaur. *Zoological Journal of the Linnean Society* **122**, 291–348.

Norman, D. B. 2002 On Asian ornithopods (Dinosauria: Ornithischia). 4. *Probactrosaurus*

Rozhdestvensky, 1966. *Zoological Journal of the Linnean Society* **136**, 113–144.

Norman, D. B. 2004 Basal Iguanodontia. In *The Dinosauria: Second Edition* (eds. D. B.

Weishampel, P. Dodson & H. Osmólska), pp. 413–437. Berkeley: University of California Press.

Norman, D. B. 2010 A taxonomy of iguanodontians (Dinosauria: Ornithopoda) from the lower

Wealden Group (Cretaceous: Valanginian) of southern England. *Zootaxa* **2489**, 47–66.

Norman, D. B. 2011 On the osteology of the lower Wealden (Valanginian) ornithopod *Barilium*

*dawsoni* (Iguanodontia: Styracosterna). *Special Papers in Palaeontology* **86**, 165–194.

Norman, D. B. & Barrett, P. M. 2002 Ornithischian dinosaurs from the Lower Cretaceous

(Berriasian) of England. *Special Papers in Palaeontology* **68**, 161–189.

Ostrom, J. H. 1961 Cranial morphology of the hadrosaurian dinosaurs of North America.

*Bulletin of the American Museum of Natural History* **122**, 33–186.

Ostrom, J. H. 1970 Stratigraphy and paleontology of the Cloverly Formation (Lower

Cretaceous) of the Bighorn Basin Area, Wyoming and Montana. *Peabody Museum Bulletin* **35**, 1–233.

Panaiotu, C. G. & Panaiotu, C. E. 2010 Palaeomagnetism of the Upper Cretaceous Sânpetru

Formation (Haţeg Basin, South Carpathians). *Palaeogeography, Palaeoclimatology,*

*Palaeoecology* **293**, 343–352.

Paul, G. S. 2008 A revised taxonomy of the iguanodont dinosaur genera and species.

*Cretaceous Research* **29**, 192–216.

Prieto-Márquez, A. 2010a *Glishades ericksoni*, a new hadrosauroid (Dinosauria: Ornithopoda)

from the Late Cretaceous of North America. *Zootaxa* **2452**, 1–17.

Prieto-Márquez, A. 2010b Global phylogeny of hadrosauridae (Dinosauria: Ornithopoda) using

parsimony and Bayesian methods. *Zoological Journal of the Linnean Society* **159**, 435–502.

Prieto-Márquez, A. & Norell, M. A. 2010 Anatomy and relationships of *Gilmoreosaurus*

*mongoliensis* (Dinosauria: Hadrosauroidea) from the Late Cretaceous of Central Asia. *American Museum Novitates* **3694**, 1–49.

Prieto-Márquez, A., Weishampel, D. B. & Horner, J. R. 2006a The dinosaur *Hadrosaurus*

*foulkii*, from the Campanian of the East Coast of North America, with a reevaluation of the genus. *Acta Palaeontologica Polonica* **51**, 77–98.

Prieto-Márquez, A., Gaete, R., Rivas, G., Galobart, À. & Boada, M. 2006b Hadrosauroid

dinosaurs from the Late Cretaceous of Spain: *Pararhabdodon isonensis* revisited and *Koutalisaurus kohlerorum*, gen. et sp. nov. *Journal of Vertebrate Paleontology* **26**, 929–943.

Rawson, P. F. 2006 Cretaceous: sea levels peak as the North Atlantic opens. In *The Geology of*

*England and Wales, 2^nd^ Edition* (eds. P. J. Brenchley & P. F. Rawson), pp. 365–394. Bath: The Geological Society of London.

Ruiz-Omeñaca, J. I. 2011 *Delapparentia turolensis* nov. gen et sp., a new iguanodontoid

dinosaur (Ornithischia: Ornithopoda) from the Lower Cretaceous of Galve (Spain). *Estudios Geológicos* **67**, 83–110.

Ruiz-Omeñaca, J. I., Pereda Suberbiola, X. & Galton, P. M. 2006 *Callovosaurus leedsi*, the

earliest dryosaurid dinosaur (Ornithischia: Euornithopoda) from the Middle Jurassic of England. In *Horns and Beaks: Ceratopsian and Ornithopod Dinosaurs* (ed. K. Carpenter), pp. 3–16. Bloomington: Indiana University Press.

Schnyder, J., Dejax, J., Keppens, E., Tu, T. T. N., Spagna, P., Boulila, S., Galbrun, B.,

Riboulleau, A., Tshibangu, J.-P. & Yans, J., 2009 An Early Cretaceous lacustrine record: organic matter and organic carbon isotopes at Bernissart (Mons Basin, Belgium). *Palaeogeography, Palaeoclimatology, Palaeoecology* **281**, 79–91.

Sereno, P. C. 1991 *Lesothosaurus*, “fabrosaurids,” and the early evolution of Ornithischia.

*Journal of Vertebrate Paleontology* **11**, 168–197.

Shibata, M., Jintasakul, P. & Azuma, Y. 2011 A new iguanodontian dinosaur from the Lower

Cretaceous Khok Kruat Formation, Nakhon Ratchasima in northeastern Thailand. *Acta Geologica Sinica-English Edition* **85**, 969–976.

Sues, H.-D. & Averianov, A. 2009 A new basal hadrosauroid dinosaur from the Late

Cretaceous of Uzbekistan and the early radiation of duck–billed dinosaurs. *Proceedings of the Royal Society B* **276**, 2549–2555.

Tang, F., Luo, Z.-X., Zhou, Z.-H., You, H.-L., Georgi, J. A., Tang, Z.-L. & Wang, X.-Z. 2001

Biostratigraphy and palaeoenvironment of the dinosaur-bearing sediments in Lower Cretaceous of Mazongshan area, Gansu Province, China. *Cretaceous Research* **22**, 115–129.

Taquet, P. 1976 Ostéologie d’*Ouranosaurus nigeriensis*, Iguanodontide du Crétacé Inférieur du

Niger. *Géologie et Paléontologie du Gisement de Gadoufaoua (Aptien du Niger)*, Chapitre III. pp. 57–168.

Taquet, P. & Russell, D. A. 1999 A massively-constructed iguanodont from Gadoufaoua,

Lower Cretaceous of Niger. *Annales de Paléontologie* **85**, 85–96.

Van Itterbeeck, J., Markevich, V. S. & Horne, D. J. 2004 The age of the dinosaur-bearing

Cretaceous sediments at Dashuiguo, Inner Mongolia, P.R. China based on charophytes, ostracods and palynomorphs. *Cretaceous Research* **25**, 391–409.

Van Itterbeeck, J., Horne, D. J., Bultynck, P. & Vandenberghe, N. 2005 Stratigraphy and

palaeoenvironment of the dinosaur-bearing Upper Cretaceous Iren Dabasu Formation, Inner Mongolia, People’s Republic of China. *Cretaceous Research* **26**, 699–725.

Wang, X., Pan, R., Butler, R. J. & Barrett, P. M. 2010 The postcranial skeleton of the

iguanodontian ornithopod *Jinzhousaurus yangi* from the Lower Cretaceous Yixian Formation of western Liaoning, China. *Earth and Environmental Science Transactions of the Royal Society of Edinburgh* **101**, 135–159.

Weishampel, D. B. & Bjork, P. R. 1989 The first indisputable remains of *Iguanodon*

(Ornithischia: Ornithopoda) from North America: *Iguanodon lakotaensis*, sp. nov. *Journal of Vertebrate Paleontology* **9**, 56–66.

Weishampel, D. B., Norman, D. B. & Grigorescu, D. 1993 *Telmatosaurus transsylvanicus*

from the Late Cretaceous of Romania: the most basal hadrosaurid dinosaur. *Palaeontology* **36**, 361–385.

Weishampel, D. B., Jianu, C.-M., Csiki, Z. & Norman, D. B. 2003 Osteology and phylogeny of

*Zalmoxes* (n. g.), an unusual euornithopod dinosaur from the latest Cretaceous of Romania.

*Journal of Systematic Palaeontology* **1**, 65–123.

Wiman, C. 1929 Die Kreide-dinosaurier aus Shantung. *Palaeontologia Sinica* **6**, 1–63.

Winkler, D. A., Murry, P. A. & Jacobs, L. L. 1997 A new species of *Tenontosaurus*

(Dinosauria: Ornithopoda) from the Early Cretaceous of Texas. *Journal of Vertebrate Paleontology* **17**, 330­­–348.

Xu X., Zhao X. J., Lu J.-C., Huang W.-B., Li Z.-Y. & Dong Z.-M. 2000 A new iguanodontian

from Sangping Formation of Neixiang, Henan and its stratigraphical implications. *Vertebrata PalAsiatica* **38**, 176–191.

You H.-L. & Li D.-Q. 2009 A new basal hadrosauriform dinosaur (Ornithischia: Iguanodontia)

from the Early Cretaceous of northwestern China. *Canadian Journal of Earth Sciences* **46**, 949–957.

You H., Ji Q. & Li D. 2005 *Lanzhousaurus magnidens* gen. et sp. nov. from Gansu Province,

China: the largest-toothed herbivorous dinosaur in the world. *Geological Bulletin of China* **24**, 785–794.

You H., Li D. & Liu W. 2011 A new hadrosauriform dinosaur from the Early Cretaceous of

Gansu Province, China. *Acta Geologica Sinica-English Edition* **85**, 51–57.

You H., Ji Q., Li J. & Li Y. 2003a A new hadrosauroid dinosaur from the mid-Cretaceous of

Liaoning, China. *Acta Geologica Sinica-English Edition* 77, 148–154.

You, H., Luo, Z., Shubin, N. H., Witmer, L. M., Tang, Z. & Tang, F. 2003b The earliest-known

duck-billed dinosaur from deposits of late Early Cretaceous age in northwest China and

hadrosaur evolution. *Cretaceous Research* **24**, 347–355.
